# Dietary meat fats and burden of cardiovascular disease risk factors, in the elderly: a report from the MEDIS study

**DOI:** 10.1186/1476-511X-9-30

**Published:** 2010-03-18

**Authors:** Evangelos Polychronopoulos, George Pounis, Vassiliki Bountziouka, Akis Zeimbekis, Ioanna Tsiligianni, Brikena-Eirini Qira, Efthimios Gotsis, George Metallinos, Christos Lionis, Demosthenes Panagiotakos

**Affiliations:** 1 Department of Nutrition Science - Dietetics, Harokopio University, Athens, Greece; 2 Health Center of Kalloni, General Hospital of Mitilini, Mitilini, Greece; 3 Clinic of Social and Family Medicine, School of Medicine, University of Crete, Heraklion, Greece

## Abstract

Dietary fats have long been associated with human health, and especially cardiovascular disease (CVD). Some observational studies have shown that reduction in dietary fats, and particularly cholesterol is associated with lower cardiovascular risk; however, other prospective studies or randomized controlled trials of dietary fat reduction or modification have shown varying results on CVD morbidity and mortality. In this work we evaluated the relationships between dietary fats and a cluster of CVD risk factors (i.e., diabetes, obesity, hypercholesterolemia, hypertension), among elderly individuals without known CVD. In particular, dietary and clinical data from 1486 elderly (aged 65 to 100 years) men and women living in Cyprus, Mitilini, Samothraki, Cephalonia, Crete, Lemnos, Syros, Naxos, Corfu and Zakynthos islands, and participated in the MEDIS study, were analysed. Data analysis revealed that 18.5% of males and 33.3% of females had three or four cardiovascular disease risk factors; the major source of fat was olive oil (mean intake for men and women 50.0 ± 19.3 g/day and 46.0 ± 16.8 g/day, p < 0.001). In addition it was observed that a 5% increase in energy adjusted fat intake from meat was associated with a 21% increase in the likelihood of having one additional CVD risk factor (95%CI 6%-39%); no significant associations were observed regarding the other types of fat consumed by the elderly participants. These findings may state a hypothesis that the consumption of fat only from meat or its products seems to increase the burden of CVD risk factors among CVD-free, elderly people.

## 

Fat intake, in any of its forms, i.e., mono-unsaturated, polyunsaturated, trans and saturated, has long being investigated in relation to human health. A number of observational studies have reported that low-fat, but high-carbohydrate consumption, is associated with reduced risk of cardiovascular disease (CVD), and particularly coronary heart disease (CHD). These facts are mainly based on the observations made in the 1960s and 1970s in populations with low intakes of saturated and total fat (like those living in the Mediterranean region, e.g., Greece) and who were also at low CVD risk [1-3]. It has already been suggested that saturated fat increases low-density lipoprotein (LDL) cholesterol levels, while high-carbohydrate reduces high-density lipoprotein (HDL) cholesterol levels and raise fasting levels of triglycerides [1]. For example, Mittendorfer and Sidossis reported that the increase in fasting plasma triglycerides in response to short-term high-carbohydrate diets is due to accelerated VLDL-triacylglycerol secretion [3]. Both LDL, HDL cholesterol have been directly associated with CVD in numerous studies [1]. However, more recently, results of prospective epidemiologic studies of dietary fat and CVD have been inconsistent. Some studies reported a positive association between dietary fat and CHD risk [4,5], but other studies failed to reach a significant result, especially when energy intake was taken into account [6,7]. Some metabolic studies suggest that the effect of dietary cholesterol on total- and LDL cholesterol levels in humans is considerably less strong than that of saturated fat [8]. As Willett reports in his textbook [9], the inconsistency of these findings could be partially attribute to the small size of some studies, the experimental design, the inadequate dietary assessment, the incomplete adjustment for energy intake, the failure to take into consideration the trans isomers of unsaturated fats, and the lack of control for other types of fat. In addition, previous research on the relation of dietary fat to the risk of CHD has focused primarily on men and middle-aged or younger adults, while data regarding the elderly are very sparse in the literature.

Therefore, the aim of the present work is to evaluate the relationship between dietary fat intake and CVD risk factors levels, among men and women older than 65 years, living in the Mediterranean islands.

## Methods

The MEDIS study [10] is a health and nutrition survey that aimed to evaluate bio-clinical, lifestyle, behavioural and dietary characteristics of 1486 elderly people living in Mediterranean islands (Cyprus Republic, *n *= 300, and Crete, *n *= 131, Lesvos, *n *= 142, Samothraki, *n *= 100, Lemnos *n *= 150, Cephalonia, *n *= 114, Corfu, *n *= 150, Zakinthos, *n *= 103, Syros, *n *= 151, and Naxos, *n *= 145, in Greece). All participants were without any clinical evidence of CVD or cancer in their medical history. A random, population-based, multistage sampling method (i.e., age group, 3 levels (65-75, 75-85, 85 ±) and 2 sex levels) was used to select men 744 (75 ± 7 years) and 742 women (73 ± 7 years). Individuals residing in assisted-living centres, as well as those with a clinical history of CVD were not included in the survey. The participation rate varied from 75% to 89%. A group of health scientists (i.e., physicians, dieticians and nurses) with previous experience in field investigation collected all the required information, using a quantitative questionnaire and standard procedures.

The retrieved data were confidential, and the study followed the ethical consideration that provided by the World Medical Association (52^nd ^WMA General Assembly, Edinburgh, Scotland, October 2000). The Institutional Reviewing Board approved the aims, design and procedures of the study. Before the interview, participants were informed about the aims and procedures of the study, and signed an informed consent.

The information included basic demographic, such as age, gender, lifestyle factors (smoking, physical activity and dietary habits), as well as various biological and clinical characteristics of the participants. Particularly, dietary habits were assessed through a semi-quantitative, validated and repeatable food-frequency questionnaire. Frequency of consumption of various food groups and beverages (i.e., meat and products, fish and fisheries, milk and other dairy, fruits, vegetables, greens and salads, legumes, cereals, coffee and tea and soft-drinks) on daily, weekly or monthly basis, was assessed. Furthermore, intake of various alcoholic beverages (i.e., wine, beer, etc.) was measured in terms of wineglasses adjusted for ethanol intake (e.g., one 100 ml glass of wine was considered to have 12% ethanol). To better evaluate overall dietary habits the MedDietScore (range 0-55) was used in order to assess the level of adherence to this traditional dietary pattern [11]. Higher values of this diet score indicates greater adherence to the Mediterranean diet. Dietary fat intake, as percent of total energy, were calculated based on exchange lists for meal planning by the American Diabetes Association and the American Dietetic Association [12], as well as the Greek Food Composition Tables [13]. Furthermore, diabetes mellitus (type 2) was determined by fasting plasma glucose tests and was analyzed in accordance with the American Diabetes Association diagnostic criteria (i.e., fasting blood glucose levels greater than 125 mg/dL or use of special medication, indicated the presence of diabetes); fasting blood lipids levels were also recorded and hypercholesterolemia was defined as total serum cholesterol levels >200 mg/dL or the use of lipid-lowering agents; participants' who had blood pressure levels ≥ 140/90 mmHg or used antihypertensive medications were classified as hypertensive; finally, weight and height were measured to attain body mass index (BMI) scores (kg/m^2^). Obesity was defined as BMI > 29.9 Kg/m^2^. A risk-factor score including diabetes, obesity, hypertension and hypercholesterolemia was developed (theoretical range 0-4) in order to measure participants' CVD risk.

Normally distributed continuous variables are presented as mean values ± standard deviation, skewed variables as median and quartiles and categorical variables as frequencies. The normality of continuous variables was tested graphically according to P-P plots. For the comparisons of dietary fats and energy intake between men and women, the independent samples t-test for normally distributed variables and Mann-Whitney test were used. Associations between dietary fats intake and CVD risk factors score was tested with Spearman's rank correlation coefficient. Ordinal multiple logistic regression analysis evaluated the association of dietary fats on the likelihood of having one additional CVD risk factor, after adjusting for various potential confounders. The proportional odds' assumption was tested graphically, while deviance residuals were calculated to evaluate model's goodness-of-fit. Results are presented as b-coefficients and their corresponding 95% confidence intervals. P-values < 0.05 from two-sided hypotheses are considered as statistically significant. All statistical calculations are performed on the SPSS version 14.0 software (SPSS Inc, Chicago, IL, U.S.A.).

## Results

**Figure 1:**
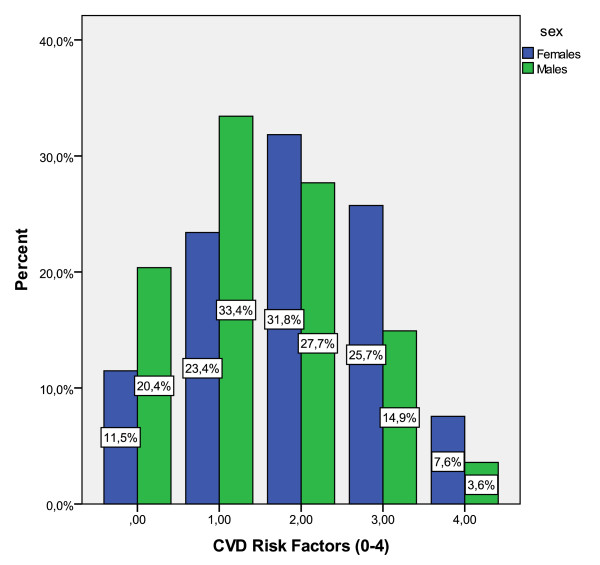
**Distribution of CVD risk factors (diabetes, obesity, hypertension and hypercholesterolemia) among 1486 elderly participants of the MEDIS study**.

In Figure 1 the distribution of CVD risk factors among the study's participants is illustrated. Interestingly, 20.4% of men and 11.5% of women had none of the investigated CVD risk factors (i.e., diabetes, hypercholesterolemia, obesity and hypertension); while, 18.5% of males and 33.3% of females had three or four of the aforementioned factors. The prevalence of CVD risk factors was higher in females compared with males (p < 0.001). Moreover, women were more likely to be obese (women vs. men: 40.6% vs. 25.0%, p < 0.001), have hypercholesterolemia (women vs. men: 59.9% vs. 41.5%, p < 0.001), and hypertension (women vs. men: 70.3% vs. 60.0%, p < 0.001), while no gender differences were observed regarding the prevalence of diabetes (women vs. men: 23.9% vs. 21.9%, p < 0.001). Concerning lifestyle characteristics, 62.1% of men and only 8.1% of women reported ever smokers (*p *for gender differences < 0.001), and 50.4% of men and 68.2% of women reported completely physically inactive (*p *for gender differences < 0.001).

Regarding dietary habits, the mean MedDietScore (i.e., approximately 32 ± 4) suggests that the level of adherence to the Mediterranean diet was 57% for males (score 31.6 × 100/55) and 58% for females (score 32.4 × 100/55). Table 1 presents the distribution of dietary fats consumed by both men and women participants. As it can be seen olive oil seems to be the main source of dietary total fat for elderly while no significant difference were observed regarding its intake between the two genders. However, men consume greater quantities of total fat and fat from fishes than women while the mean and median daily intake were 50.0 ± 19.3 g, 2.9 g (1.4-4.2 g) and 46.0 ± 16.8 g, 2.7 g (1.2-3.6 g) correspondingly (*p *for all comparisons < 0.05). Unadjusted analysis was applied in order to examine the correlation between consumption of different types of dietary fat and the CVD risk factors' score. The results showed that total fat consumption and fat intake from meat as percents of total energy consumption were positively correlated with the number of CVD risk factors (Spearman's rho = 0.16, *p *< 0.001 and Spearman's rho = 0.06, *p *= 0.05, respectively). In contrary, fat intake from foods high in carbohydrates (legumes, pasta, potatoes and cereals) were inversely correlated with CVD risk score (Spearman's rho = -0.07, *p *= 0.02).

**Table 1:** Dietary fat distribution in elderly men and women from the MEDIS study.

	Men	Women	P*
N	744	742	
Energy intake (kcal/day)	1702 ± 638	1654 ± 574	0.17
Total fat intake (g/day)	50.0 ± 19.3	46.0 ± 16.8	< 0.001
Percent of total fat (%Energy)	35.2 ± 6.5	35.3 ± 6.0	0.80
Percent of fat from			
*meat (%Energy)*	5.7 (4.4-9.9)	6.0 (4.6-10.7)	0.13
*fish (%Energy)*	2.9 (1.4-4.2)	2.7 (1.2-3.6)	0.01
*sweets (%Energy)*	1.7 (0.7-6.0)	2.2 (0.8-6.2)	0.09
*olive oil (%Energy)*	22.2 (16.3-26.9)	22.5(17.0-26.9)	0.50
high carbohydrate food groups** (%Energy)	3.5 ± 1.2	3.4 ± 1.2	0.10

Data are presented as mean ± standard deviation or median and quartiles (25^th ^- 75^th^).

* P-values were derived through independent samples t-test for normally distributed variables and Mann-Whitney test for skewed.

** Legumes, pasta, potatoes, cereals

However, residual confounding may exists, thus, after adjusting for age, gender, body mass index, MedDietScore, smoking habits and physical activity status, data analysis revealed that only fat intake from meat was positively associated with likelihood of having one additional CVD risk factor (Table 2). Specifically, a 5% energy adjusted increase in fat intake from meat is associated with 21% increase in this likelihood of having one additional CVD risk factor (95%CI 6% - 39%). However, the mean effect of total fat intake or fat consumption from other different foods groups on the likelihood of having one additional CVD risk factor was non-significant (*p *for all > 0.05).

**Table 2:** Results from multi-adjusted ordinal logistic regression analysis that evaluated the association between dietary fats intake (independent factors) and the level of CVD risk factors (dependent outcome) among elderly men and women (the MEDIS study).

Independent factors	Odds ratio for 5% increase	95% confidence interval
*Model 1*: total fat (%Energy)	1.00	0.92 - 1.10
*Model 2*: fat from meat (%Energy)	1.21	1.06 - 1.39
*Model 3*: fat from fish (%Energy)	0.94	0.75 - 1.17
*Model 4*: fat from high carbohydrates foods (%Energy)	0.81	0.51 - 1.29
*Model 5*: fat from sweets (%Energy)	0.93	0.84 - 1.03
*Model 6*: fat from olive oil (%Energy)	1.03	0.97 - 1.09

All models have been adjusted for age, sex, body mass index, MedDietScore, smoking habits and physical activity status.

## Discussion

This survey evaluated the association of dietary fat with the presence of diabetes, hypercholesterolemia, obesity and hypertension among elderly people living in Mediterranean islands. Data analysis revealed that the burden of CVD factors, among CVD-free elderly people, was moderate, while only 18.5% of elderly males and 33.3% of elderly females had three or four of the aforementioned factors. Moreover, our results may suggest that only fat consumption from meat and its products is positively associated with the likelihood of having increased CVD risk factors burden, while fat intake from any other source had no significant effect.

The observed prevalence of CVD risk factors among elderly persons of our sample may be partially explained by the shift toward less healthy dietary habits in recent decades. Recent studies, especially in Europe, have shown strong evidence that the Mediterranean dietary pattern observed during the 1950 s and 1960 s has changed to a more "Westernized" type of diet for individuals of all ages [14,15]. In Greece, evidence from the ATTICA and other epidemiologic studies has already suggested that the eating habits of young and middle-aged have changed to a more "westernized" pattern [15] while previous reports of the MEDIS study, studies by Kafatos et al., and the 350 EPIC-elderly group [14,16] had similar results. In the present sample adherence to the Mediterranean diet was moderate 57% in males thus it is not surprising that the high rates of diabetes, hypercholesterolemia, obesity and hypertension prevail especially in women. In addition, several studies suggest that increased total and saturated fat consumption is associated with higher CVD risk. In a large cohort of 43.757 health professionals in US aged 40 to 75 years free of diagnosed cardiovascular disease, was observed that high saturated fat consumption was associated with 22% increase in risk of CVD-event in 10-years of follow-up [5]. Moreover, in another prospective study of 1001 middle-aged men after 20 years of follow-up it was suggested that high saturated fat intake is associated with 60% higher risk of coronary heart disease [4]. Our findings is somehow in accordance to these evidence while in the present work is indicated that 5% increase in fat intake from meat as a percent of total energy consumption is associated with 21% increase in the likelihood of having one another CVD risk factor. As it is well known fat from meat and its products contain high proportions of saturated lipids which are strongly associated with the prevalence of several risk factors for heart disease. In addition, saturated fat increases low-density lipoprotein (LDL) cholesterol levels which has implications to the functioning of the endothelium [1,17]. Saturated fatty acids along with trans-fatty acids and cholesterol also interfere with EFA metabolism and promote inflammation, atherosclerosis and coronary heart disease [18-20]. They actually contribute to the formation of Pro-inflammatory molecules and consequently to the Low grade systematic inflammatory conditions. This is a possible biological explanation of how fat from meat and its products may affect the likelihood of having more CVD risk factors [21-23].

In our sample there are not gender differences concerning the meat intake. The MEDIS elderly participants seem to be moderate meat-eaters, as also a "typical subsample" of adult male Australians Study [20]. According to this study regular or moderate consumption of meat and fish maintains a plasma FA profile possibly more conducive to good health. Moreover, from a previous study of Li D et al., revealed that dietary intake of PUFA and fish are potential confounding factors for assessing the effects of meat consumption on platelet individual PUFA [21]. In our study the association of total fat intake or fat consumption from other different foods groups of having one additional CVD risk factor was not significant. Probably alternate meat and fish intake facilitates the balance of homeostatic end physiological pathways.

## Limitations

This study is cross-sectional and consequently, has the potential of recall biases, particularly in the assessment of dietary habits. Although important associations were assessed, the design of this study prohibits causal interpretations. In our sample we have also no specific data on the type of meat (i.e., pig meat, beef, sheep, lambs, rabbit, poultry, or wild animals). Regarding trans fatty acids intake we do not have exact data in MEDIS sample, but according to the TRANSFAIR Study [24] in late 1990s it was considered that trans-fatty acids intake was lowest in Mediterranean countries, like Greece and Italy (i.e., 0.5%-0.8%), compared with other countries around the world.

## Conclusions

Under the context of the MEDIS Study the present work evaluated the association of dietary fat intake on CVD risk factors, based on a large sample of elderly men and women living in Mediterranean islands; it was revealed that high intake of fat from meat was associated with an increased likelihood of having more CVD risk factors among the elderly. According to previous studies meat is not an essential component of the diet and societies that have adopted vegetarian studies for religious or other reasons do not show any evidence of malnutrition when the supply of total food is adequate. Apparently meat is conventionally considered as a protein food and an important source of fat. On the contrary, high meat consumption highlights a crucial important public health issue, especially in the elderly. It is of great importance that access to primary health care and prevention services in the investigated isolated regions is low and may enhance the problem. Furthermore there is a scientific hope with the restructured meat products with added wholesome ingredients (i.e., walnuts), which can be considered functional foods with cholesterol-lowering effect for subjects at high risk for CVD [25].

## Competing interests

The authors declare that they have no competing interests.

## Authors' contributions

EP: wrote the paper and contributed in the design of the study, GP: wrote the paper and participated in the enrolment of the participants, VB, AZ, IT, BEQ, EG, GM, CL local coordinators, participated in the enrolment of the participants, and reviewed the paper, DP: designed and coordinated the study, supervised the data analyses, critically reviewed the paper. All authors read and approved the final manuscript.
